# Molecular Characterization of Pancreatic Ductal Adenocarcinoma Using a Next-Generation Sequencing Custom-Designed Multigene Panel

**DOI:** 10.3390/diagnostics12051058

**Published:** 2022-04-23

**Authors:** Deborah Malvi, Francesco Vasuri, Thais Maloberti, Viviana Sanza, Antonio De Leo, Adele Fornelli, Michele Masetti, Claudia Benini, Raffaele Lombardi, Maria Fortuna Offi, Mariacristina Di Marco, Matteo Ravaioli, Sirio Fiorino, Enrico Franceschi, Alba A. Brandes, Elio Jovine, Antonietta D’Errico, Giovanni Tallini, Dario de Biase

**Affiliations:** 1 Pathology Unit, IRCCS, Azienda Ospedaliero-Universitaria di Bologna, 40138 Bologna, Italy; deborah.malvi@aosp.bo.it (D.M.); francesco.vasuri@aosp.bo.it (F.V.); antonietta.derrico@unibo.it (A.D.); 2 Department of Experimental, Diagnostic and Specialty Medicine, University of Bologna-Molecular Diagnostic Unit, Azienda USL di Bologna, 40138 Bologna, Italy; thais.maloberti2@unibo.it (T.M.); antonio.deleo@unibo.it (A.D.L.); giovanni.tallini@ausl.bologna.it (G.T.); 3 Division of Molecular Pathology, IRCCS, Azienda Ospedaliero-Universitaria di Bologna, 40138 Bologna, Italy; viviana.sanza@ausl.bologna.it; 4 Anatomic Pathology Unit, Azienda USL, Maggiore Hospital, 40133 Bologna, Italy; adele.fornelli@ausl.bologna.it; 5 Department of General Surgery, IRCCS, Azienda Ospedaliero-Universitaria di Bologna, Maggiore Hospital, 40133 Bologna, Italy; m.masetti@ausl.bologna.it (M.M.); claudia.benini@ausl.bologna.it (C.B.); raffaele.lombardi@ausl.bologna.it (R.L.); mariafortuna.offi@ausl.bologna.it (M.F.O.); elio.jovine@ausl.bologna.it (E.J.); 6 Division of Medical Oncology, IRCCS, Azienda Ospedaliero-Universitaria di Bologna, 40138 Bologna, Italy; mariacristina.dimarco@unibo.it; 7 Department of Specialized, Experimental and Diagnostic Medicine, University of Bologna, 40138 Bologna, Italy; 8 Department of General Surgery and Transplantation, IRCCS, Azienda Ospedaliero-Universitaria di Bologna, 40138 Bologna, Italy; matteo.ravaioli6@unibo.it; 9 Dipartimento di Scienze Mediche e Chirurgiche (DIMEC), University of Bologna, 40126 Bologna, Italy; 10 Internal Medicine Unit, Budrio Hospital, Budrio (Bologna), Azienda USL di Bologna, 40054 Bologna, Italy; sirio.fiorino@ausl.bologna.it; 11 Nervous System Medical Oncology Department, IRCSS Istituto di Scienze Neurologiche di Bologna, 40139 Bologna, Italy; enricofra@yahoo.it (E.F.); alba.brandes@yahoo.it (A.A.B.); 12 Department of Pharmacy and Biotechnology (FaBiT), University of Bologna, 40138 Bologna, Italy

**Keywords:** pancreatic cancer, PDAC, next-generation sequencing, *KRAS*, *TP53*, mutations

## Abstract

Despite the efforts made in the management of PDAC, the 5-year relative survival rate of pancreatic ductal adenocarcinoma (PDAC) still remains very low (10%). To date, precision oncology is far from being ready to be applied in cases of PDAC, although studies exploring the molecular and genetic alterations have been conducted, and the genomic landscape of PDAC has been characterized. This study aimed to apply a next-generation sequencing (NGS) laboratory-developed multigene panel to PDAC samples to find molecular alterations that could be associated with histopathological features and clinical outcomes. A total of 68 PDACs were characterized by using a laboratory-developed multigene NGS panel. *KRAS* and *TP53* mutations were the more frequent alterations in 75.0% and 44.6% of cases, respectively. In the majority (58.7%) of specimens, more than one mutation was detected, mainly in *KRAS* and *TP53* genes. *KRAS* mutation was significantly associated with a shorter time in tumor recurrence compared with *KRAS* wild-type tumors. Intriguingly, *KRAS* wild-type cases had a better short-term prognosis despite the lymph node status. In conclusion, our work highlights that the combination of *KRAS* mutation with the age of the patient and the lymph node status may help in predicting the outcome in PDAC patients.

## 1. Introduction

Pancreatic ductal adenocarcinoma (PDAC) still represents the major cause of death in Western countries. Despite all the efforts made regarding the comprehension of the cancerogenesis of PDAC and toward its genomic key alterations, the 5-year survival rate of these patients is still very low (below 10%) [1,2] and, unfortunately, only a minority are suitable for resection at the time of the diagnosis [3]. Several efforts have been performed in the management of the PDAC with advances in the surgical, as well as the oncological fields and its multimodal approach, but the prognosis remains poor and has not significantly changed in the last decades [3]. To date, personalized therapy is at the basis of the modern concept of oncological treatments and represents the precision medicine that is able to target different specific proteins that control cancer growth, apoptosis, and or spread in every single patient. These results have been obtained thanks to the advances in molecular biology coupled with improvements in laboratory technology devices that have led to the deepest insight into tumor biology. Currently, many types of tumors are treated with targeted approaches with several choice drugs (such as gastrointestinal stromal tumors, melanoma, breast tumors, ovarian cancers, colorectal cancers, and lung cancers [4,5,6]).

Several papers have demonstrated the association between clinicopathological features, such as lymph node spread and positive surgical margins, and a worse outcome [7,8,9,10]; however, these characteristics have been found also in 20–40% of patients with a good prognosis [7,8,9,10], suggesting that clinicopathological features are not the only determinant for predicting PDAC prognosis. Precision oncology is far from being ready to be applied in cases of PDAC, although studies exploring the molecular and genetic alterations have been conducted, and the genomic landscape of PDAC has been characterized [11,12,13]. Furthermore, relatively few clinically drugable mutations have been identified, and there are still not any targeted agents against the four major genes altered in PDAC that have been validated. In fact, to date, no targetable molecules are available for personalized treatment in the clinical practice of PDAC, as stated by the current ESMO (European Society for Medical Oncology) guidelines [14,15]. Different types of molecular alterations may be found in PDAC, from oncogenes mutations to inactivation of tumor suppressor genes/genes controlling the repair of DNA damage [16]. The most frequently involved and mutated genes in PDAC tumorigenesis are early events in cancerogenesis, such as *KRAS* mutations or CDKN2A/p16 loss of function, and alterations occurring later in the neoplastic progression, such as *TP53* mutations and SMAD4/DPC4 loss [17,18,19,20,21]. Other alterations involved with less frequency have been identified, such as those in the mismatch repair deficiency (dMMR) genes (accounting for about 1–2% of PDAC), *NTRK* fusions (about 0.3%), or *BRCA1/2* mutations (5–10%) [5,16,21,22,23].

Previous studies have demonstrated a worse prognosis in PDAC harboring mutations in *KRAS*, *TP53*, *CDKN2A*, or *SMAD4* genes [5,22,23,24,25,26]. In particular, *KRAS* mutations have been associated with a decreased overall survival (OS), and *TP53* alterations have been associated with a poor prognosis [12,24,25].

The aim of this study was to apply an NGS laboratory-developed multigene panel to treatment-naive PDAC samples in order to find any molecular alterations that could be associated with histopathological features and clinical outcomes helping in the choice of patient clinical management.

## 2. Materials and Methods

### 2.1. Case Selection

A total of 68 PDAC samples were retrieved from the archives of the Anatomic Pathology of Maggiore Hospital (Bologna, Italy) and the Molecular Pathology Laboratory of Bologna (Bologna, Italy).

We retrospectively analyzed a total of 68 cases of treatment-naive PDAC that underwent surgical resection upfront. The cases had been collected from the pathological archives of the Molecular Pathology Laboratory (Bologna, Italy): 35 patients underwent duodenocephalopancreasectomy, 20 patients had a total pancreatectomy, and in 13 patients a distal pancreatic resection was performed. All patients underwent computed tomography (CT) scan of the whole body, with contrast material, to evaluate the possible presence of metastases and the resectability of the tumor. Moreover, CT allowed for the evaluation of the presence of neoplastic dissemination in mesenteric and splenic vessels. In all cases, a histological diagnosis of ductal pancreatic adenocarcinoma had been performed.

All cases had been revised by a GI-dedicated pathologist (DM and AF), blind to each other, to collect the main histological variable (i.e., grading, lymph vascular invasion, perineural invasion, surgical margins, and lymph node metastases) and to determine the pathological stage according to the 8th Edition of AJCC (American Joint Committee on Cancer) [27]. In controversial cases, a definitive diagnosis was made after a collegial discussion and joint agreement.

In order to identify the main representative neoplastic area for NGS analysis, the tumor area was marked on the control slide, and the proportion of neoplastic cells vs. non-neoplastic cells (i.e., endothelial, stromal, and inflammatory cells) in the area marked on the slide and used for DNA extraction was estimated after microscopic evaluation, providing the tumor cell enrichment (i.e., neoplastic cells/total number of cells).

The mean follow-up was 44.2 + 54.3 months. At the end of the available follow-up, 32 of 64 (50.0%) died. Twenty-three of 32 (71.9%) died within the first 24 months after the date of surgery. Nineteen patients (29.7%) were still alive 60 months after the date of surgery.

### 2.2. DNA Extraction and Next-Generation Sequencing Analysis

For each sample, a pathologist (AF and DM) evaluated the most representative neoplastic area, and DNA was extracted starting from two or three 10 µm–thick sections, using the “QuickExtract™ FFPE DNA Extraction Solution” kit (Lucigen Corporation, Middleton, WI, USA), and quantified by using Qubit dsDNA BR Assay Kit (Thermo Fisher Scientific, Waltham, MA, USA). An “extended version” of a previously published NGS lab-developed multigene panel [28] was used. The “extended version” of the panel includes the following genomic regions (human reference sequence hg19/GRCh37, total of 343 amplicons, 21.77 kb): *BRAF* (exons 11, 15), *c-Kit* (exons 8, 9, 11, 13, 14, 17), *CTNNB1* (exons 3, 7, 8), *DICER1* (exons 10, 21, 26, 27, 29), *DPYD* (exons 11, 13, 22, chr1 intronic regions: g.98187018- 98187098, g.98045419- 98045499, g.97915570-97915789), *EGFR* (exons 18, 19, 20, 21), *EIF1AX* (exons 1, 2, and chrX intronic region g.20148634–20148745), *GNA11* (exons 4, 5), *GNAQ* (exons 4, 5), *GNAS* (exons 8, 9), *H3F3A* (exon 1), *HRAS* (exons 2–4), *IDH1* (exon 4), *IDH2* (exon 4), *KRAS* (exons 2–4), *MED12* (exons 1, 2), *MET* (exons 2, 14), *NRAS* (exons 2–4), *PDGFRα* (exons 12, 14, 18), *PIK3CA* (exons 8, 10, 21), *PTEN* (exon 5), *RET* (exons 5, 8, 10, 11, 13, 15, 16), *RNF43* (exons 2–10), *SMAD4* (exons 2–12), *TERT* (promoter region, Chr5 g.1295141-1295471), and *TP53* (exons 2–11), *TSHR* (exons 1–10), *VHL* (exons 1–3). Briefly, about 50 ng of input DNA was used for NGS libraries preparation with the AmpliSeq Plus Library Kit 2.0 (Thermo Fisher Scientific). Templates were then sequenced by using an Ion 530 chip, and the results were analyzed with the IonReporter tools (version 5.16, Thermo Fisher Scientific) and IGV software (Integrative Genome Viewer version 2.9.2—https://software.broadinstitute.org/software/igv/, accessed on 22 December 2021). According to the previously reported validation [28], only mutations present in at least 5% of the total number of reads analyzed and observed in both strands were considered for mutational calls. The significance of alterations was checked by using the Varsome database (https://varsome.com/, accessed on 22 December 2021) and “IARC TP53 Database” (http://p53.iarc.fr/, accessed on 22 December 2021).

### 2.3. Statistical Analysis

In this study, we chose to evaluate as our final end-point the occurrence of the death of the patients as the major event. The *p*-values less than 0.05 were considered statistically significant. Variables that were found to be significant on univariate analysis at *p* < 0.1 were included in multivariate analysis in a backward stepwise fashion. Cox proportional hazards models were generated for multivariate analysis. Statistical analysis was performed by using SPSS Statistics 20.

## 3. Results

Clinicopathological and molecular characteristics of the cohort analyzed are reported in Table 1.

### 3.1. Molecular Alteration in PDAC

DNA was amplifiable in 64 of 68 samples (94.1%), while, in four cases, the quality/quantity of DNA was not enough for us to obtain reliable results. In these cases, the coverage obtained after the NGS analysis was too low (<100 reads per amplicon), and then the samples were excluded from the following analyses. This inadequacy of the specimens may be due to an over-degradation of the samples as a result of pre-analytic conditions (e.g., prolonged formalin fixation). At least one gene alteration was detected in 52 of 64 cases (81.2%), while, in 12 cases (18.8%), no alterations were detected the in genes analyzed.

**Figure 1:**
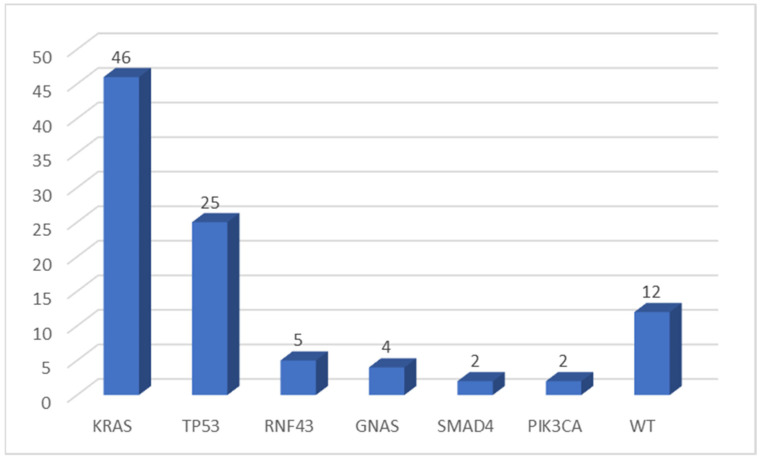
Number of mutations detected by multigene NGS panel. WT, wild type.

*KRAS* was the most frequently mutated gene (46 of 64 evaluable samples, 71.9%), while *TP53* was altered in 25 cases (39.0%). The more frequent *KRAS* mutation was the p.Gly12Asp substitution (34.8% of *KRAS* mutated samples). No p.Gly12Cys mutation was detected in our cohort. Other genes were found to be mutated in a few numbers of cases: *RNF43* in 5 of 64 (7.8%), *GNAS* in 4 (6.3%), *SMAD4* in 2 samples (3.1%), and *PIK3CA* altered in two tumors (3.1%) (Figure 1). All variants found in this cohort of pancreatic tumors were pathogenic mutations (according to the Varsome database), except for one *TP53* mutation, two *RNF43* variants, and two *SMAD4* alterations (classified as VUS—variant of uncertain significance).

#### Concomitant Mutations in PDAC

**Figure 2:**
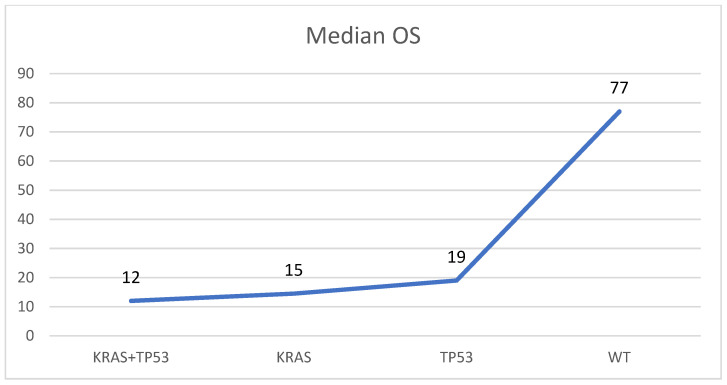
Median overall survival (OS) in patients with PDAC without *KRAS*/*TP53* mutations (WT), with a mutation in *KRAS* or *TP53*, and with mutations in *KRAS* and *TP53* (KRAS + TP53). Months are reported on the *Y*-axis.

In 28 of 52 mutated samples (53.8%), concomitant alterations were detected. The most frequent combination was between *KRAS* and *TP53*, found altered together in 20 of 52 mutated specimens (28.5%). In four samples (8.7%) another two alterations were found (one *KRAS* + *GNAS*, two *KRAS* + *RNF43*, and one *KRAS* + *SMAD4*). In three samples (4.3%) three concomitant mutations were observed (one *KRAS* + *GNAS* + *RNF43*, one *KRAS* + *TP53* + *RNF43*, and one *KRAS* + *RNF43* + *PIK3CA*), and in one specimen (2.2%), four variants were observed (*KRAS* + *GNAS* + *SMAD4* + *PIK3CA*). In the remnant 24 samples, single alterations were observed: 18 samples (75.0%) harbored a single *KRAS* mutation, 4 (16.7%) harbored only *TP53* variants, and 2 cases (8.3%) contained one *GNAS* alteration. The median overall survival (OS) was higher in patients with PDAC harboring no *KRAS*/*TP53* mutations (77 months) if compared to those with *TP53* (OS: 19 ms), *KRAS* (OS: 15 ms), or *KRAS* and *TP53* (OS: 12 ms) mutations (Figure 2).

### 3.2. Correlation between Molecular/Clinicopathological Features and Outcome

As far as the histopathological variable is concerned, the tumor stage (represented by tumor size and lymph node status), as well as perineural invasion, vascular invasion, and margin resection status, is a well-known predictive marker of recurrence and seems to be related to survival in PDCA patients [29,30,31,32,33].

**Figure 3:**
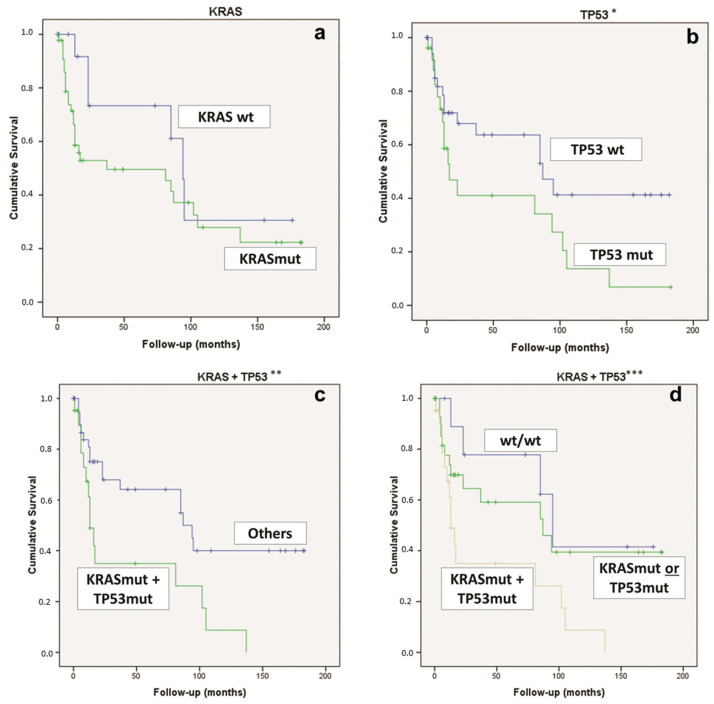
Kaplan–Meier curve comparing (**a**) PDAC *KRAS*-WT vs. *KRAS*-mut (*p* = 0.151); (**b**) PDAC *TP53*-WT vs. *TP53*-mut (* *p* = 0.037); (**c**) PDAC *KRAS* & *TP53*-mut vs. “other” PDAC (** *p* = 0.023); (**d**) PDAC *KRAS* & *TP53*-mut vs. *KRAS*-mut or *TP53*-mut vs. *KRAS*-WT and *TP53*-WT (*** *p* = 0.016). mut: mutated; WT, wild type.

Considering the whole overall survival, for the univariate analysis, the statistically significant features were *TP53* mutations (*p* = 0.037) and the concomitant *KRAS* & *TP53* mutations (*p* = 0.023) (Figure 3). On the contrary, the *KRAS* mutation alone was not statistically significant (Figure 3).

We evaluated the clinicopathological and molecular features of the patients who died within the first 24 months after surgery (*n* = 23 patients—71.9%). Of these 23 PDAC patients, 21 (91.3%) harbored *KRAS* mutations (*p* = 0.021), 13 (56.5%) had a *TP53* mutation (*p* = 0.047), and 12 (52.2%) had concomitant *KRAS* and *TP53* alterations (*p* = 0.031) (Table 2). Intriguingly, 15 PDACs (65.2%) showed perineural invasion (*p* = 0.028).

Taking into consideration the 19 patients with PDAC who were still alive after 60 months, in concordance with data reported in the literature, we found that these patients, compared with those with overall survival of fewer than 60 months, showed the following: (i) fewer incidences of vascular invasion (52.6% vs. 91.7%, respectively, *p* = 0.018) and (ii) a lower incidence of lymph-nodal involvement (36.8% vs. 81.8%, *p* = 0.001).

As confirmation, at logistic regression, a multivariate analysis considering only the histopathological variables of the lymph-nodal involvement was the most important parameter toward cancer-related death (Exp(B) = 1.349).

Including also molecular characterization of PDAC, the strongest feature was the presence of concomitant *KRAS* & *TP53* mutations (Exp(B) = 1.600).

**Figure 4:**
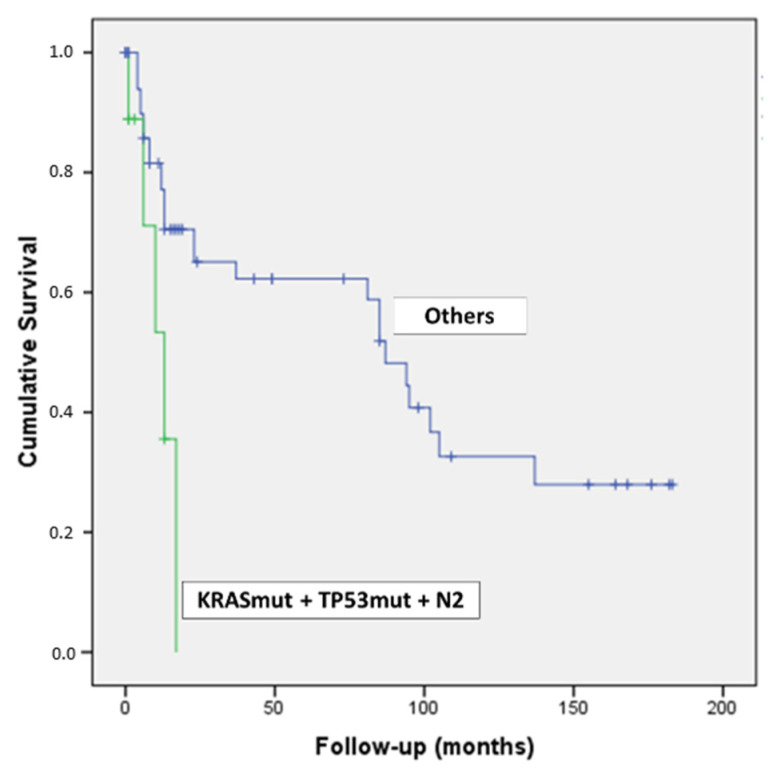
Kaplan–Meier curve of patients with PDAC harboring *KRAS* & *TP53* mutations and N2 lymph-nodal status.

Intriguingly, by combining the concomitant presence of *KRAS* & *TP53* mutations and N2 lymph-nodal status, we detected nine patients with a dramatic outcome (*p* = 0.007) (Figure 4).

## 4. Discussion

Pancreatic ductal adenocarcinoma (PDAC) is usually associated with a poor prognosis and a low survival rate, less than 10% at five years [12]. Despite all the efforts in these years to investigate and study the genetic and molecular alterations in pancreatic cancer, the majority of patients present with locally advanced or metastatic disease at the time of the diagnosis [12], and there are not still powerful druggable mutations that could be used in the clinical practice.

The characterization of multiple markers in solid tumors, using the NGS approach, has allowed for the translation of several molecular alterations into the clinical practice of metastatic tumors, as in lung adenocarcinomas, colorectal cancers, prostate cancer, ovarian carcinoma, and cholangiocarcinomas [34]. The clinical significance of molecular alterations found in pancreatic ductal adenocarcinoma is still controversial. PDACs are usually characterized by *KRAS* mutations, *TP53* alterations, and/or loss of SMAD4 or CDKN2A/p16 proteins [11,12,13,17,18,19,20,21].

In this study, we tested a multigene laboratory-developed NGS panel used to characterize PDAC in daily clinical practice. Herein, we wanted to investigate the utility of using a laboratory-developed multigene panel for the molecular characterization of PDAC, aiming to identify prognostic biomarkers to improve the clinical management of PDAC patients.

Our data confirm that *KRAS* and *TP53* are the most common genes altered in a series of PDACs, as previously described in the literature [23,25,28,35]. Other mutations (i.e., *RNF43*, *GNAS*, *SMAD4*, and *PIK3CA*) have been rarely found in our cohort. As previously reported [24], several PDAC samples showed concomitant alterations, mainly in *KRAS* and *TP53* genes.

In our study, we demonstrated that PDCA patients’ survival is related to the presence of the *TP53* or *KRAS* mutation: the presence of PDAC harboring *TP53* mutations worsens the survival if compared to the *TP53* wild-type tumors. Even if *KRAS* mutations are not significantly associated with overall survival, it should be considered that it seems to affect the outcome of the patients in the first part of the survival curve (Figure 3). Considering that patients died within the first 24 months from surgery, almost all (91.3%) harbored a *KRAS* mutation, more than half (56.5%) of them had a *TP53* alteration, and concomitant *KRAS* and *TP53* alterations were detected in about a half (52.2%). Intriguingly, only two patients who died within 24 months had a PDAC without *KRAS* and/or *TP53* alterations. Moreover, patients with PDAC harboring the concomitant mutations in *KRAS* and *TP53* have a significantly worse OS if compared to those with PDAC, with only one of the two genes mutated, or with both *KRAS* and *TP53* wild-type (Figure 3).

Analyzing the clinicopathological features, we see that the only statistically significant variable in a logistic regression multivariate analysis was represented by the lymph-nodal status. The other histological parameters that the literature reported to be predictive of recurrence and to be related to survival in PDCA patients (i.e., the tumor stage, the presence of perineural or vascular invasion, and the margin resection status) did not demonstrate a significant prognostic value in term of OS in our patient cohort.

Combining the *KRAS* and *TP53* double-mutated tumor phenotype with the abovementioned lymph-nodal stage at the time of surgery, we observed a dramatic prognosis in the double-mutated PDAC patients with an N2 stage compared to all the other patients (wild-type phenotype or tumors harboring just one mutation, without lymph-nodal spread, N0, or with limited ones, N1) (Figure 4).

Based on our NGS analysis, the current study confirms the data reported by the literature about the main driver genes (i.e., *KRAS* and *TP53*) found mutated in PDAC.

The *KRAS* p.G12C variant is a mutation that could be targeted by specific inhibitor molecules [36]. However, we have not found the *KRAS* p.G12C mutation in our cohort of patients.

In 2020, the ESMO outlined the indications for the use of NGS in the characterization of several metastatic cancers, including pancreatic ductal adenocarcinoma. According to these guidelines, it is not currently recommended to perform multigene NGS in daily practice, even if multigene sequencing is encouraged in order to get access to innovative drugs [34]. Moreover, NGS can be an alternative technique to PCR-based assays if it is not associated with extra costs for the public healthcare system and if the patient is informed about the putative benefits of this analysis [34]. Our results confirm that, in daily practice testing, *KRAS* and *TP53* alone may be sufficient to improve the clinical management of the patients, and that NGS multigene panels can be used in clinical research centers to increase access to innovative clinical study. Moreover, our data highlight that PDACs with *KRAS* and *TP53* wild-type may represent a distinct molecular subtype of pancreatic cancer that could benefit from tailored treatments [37]. This double-negative PDAC may also be investigated for the presence of HRR genes alterations, detecting a putative cohort of patients that could benefit from PARP inhibitors treatment. On the contrary, the analysis of *KRAS* and *TP53* genes in PDAC patients would help to identify those subjects with worse overall survival (i.e., *KRAS* and *TP53* mutated PDAC).

## 5. Conclusions

In conclusion, our work highlights that PDCAs harboring both *KRAS* and *TP53* mutation have a shorter OS compared to wild-type tumors or PDCAs harboring only one mutation. The presence of a high lymph-nodal patient’s stage at the time of surgery, combined with the presence of a double mutated tumor phenotype, is indicative of a dramatically short OS.

A clinical implication that can be gathered from our study is the possibility to translate the knowledge of *KRAS* and *TP53* mutational status pre-operatively from cytological material and fine-needle biopsy tissue, conditioning the future clinical management of patients with PDAC. It should be considered that the recent improvement in the needles for endoscopic ultrasound-guided fine-needle biopsy (EUS-FNB) has allowed us to obtain a large amount of material also in preoperative procedures, providing enough cells for molecular analysis in pancreatic lesions [38,39]. Moreover, a good concordance between EUS-FNB and surgical (both formalin-fixed paraffin-embedded) specimens has been demonstrated [40]. For these reasons, the analysis of *KRAS* and *TP53*, using NGS or other molecular techniques, could be easily used also in preoperative specimens. The molecular analyses performed on preoperative material of pancreatic lesions, together with the lymph node status, may help to hypothesize patients’ outcomes and influence the decision-making process, for example, pushing toward neoadjuvant chemotherapy.

## Figures and Tables

**Table 1:** Clinicopathological and molecular characteristics of the cohort analyzed by NSG.

Clinicopathological Characteristics	Number of Samples
Total cohort	68
Cases with DNA evaluable for NGS analysis	64 (94.1%)
Male	34 (53.1%)
Female	30 (46.9%)
Mean age (range)	65.8 ± 9.5 years (44–84 years)
Lymph-nodal status	
N0	27 (42.2%)
N1	15 (23.4%)
N2	22 (34.4%)
Mean size, mm (range)	29.3 ± 14.7 (10–70)
pT1	16 (25.0%)
pT2	26 (40.6%)
pT3	22 (34.4%)
Margins status	
R0	46 (71.9%)
R1	18 (28.1%)
Histological grade	
2	22 (32.1%)
3	39 (57.2%)
4	3 (1.8%)
Vascular invasion	
Yes	43 (67.2%)
No	21 (32.8%)
Perineural invasion	
Yes	41 (64.1%)
No	23 (35.9%)
DNA evaluable for NGS analysis	64 (94.1%)
Samples mutated in at least 1 gene	52 (81.2%)
Samples WT	12 (18.8%)
*KRAS*	46 (71.9%)
*TP53*	25 (39.0%)
Other mutated genes	11 (17.2%)

NGS, next-generation sequencing; WT, wild type.

**Table 2:** Molecular characteristics of patients who died within 24 months compared to those of patients with OS higher than 24 months.

Features	Number of Cases with OS < 24 months (*n* = 23)	Number of Cases with OS > 24 months (*n* = 41)	*p*-Value
*KRAS* mutation			*p* = 0.021
Yes	21 (91.3%)	14 (34.1%)	
No	2 (8.7%)	27 (65.9%)	
*TP53* mutation			*p* = 0.047
Yes	13 (56.5%)	13 (31.7%)	
No	10 (43.5%)	28 (68.3%)	
*KRAS* & *TP53* mutations			*p* = 0.031
*-KRAS* & *TP53*	12 (52.2%)	9 (22.0%)	
*KRAS* or *TP53*	9 (39.1%)	21 (51.2%)	
WT/WT	2 (8.7%)	11 (26.8%)	

## Data Availability

All available data are contained within the article.

## References

[1] Siegel R.L., Miller K.D., Fuchs H.E., Jemal A. (2021). Cancer Statistics, 2021. CA Cancer J. Clin..

[2] Cao L., Huang C., Cui Zhou D., Hu Y., Lih T.M., Savage S.R., Krug K., Clark D.J., Schnaubelt M., Chen L. (2021). Proteogenomic characterization of pancreatic ductal adenocarcinoma. Cell.

[3] Hidalgo M. (2010). Pancreatic cancer. N. Engl. J. Med..

[4] Moore M.J., Goldstein D., Hamm J., Figer A., Hecht J.R., Gallinger S., Au H.J., Murawa P., Walde D., Wolff R.A. (2007). Erlotinib plus gemcitabine compared with gemcitabine alone in patients with advanced pancreatic cancer: A phase III trial of the National Cancer Institute of Canada Clinical Trials Group. J. Clin. Oncol..

[5] Qian Y., Gong Y., Fan Z., Luo G., Huang Q., Deng S., Cheng H., Jin K., Ni Q., Yu X. (2020). Molecular alterations and targeted therapy in pancreatic ductal adenocarcinoma. J. Hematol. Oncol..

[6] Sinn M., Bahra M., Liersch T., Gellert K., Messmann H., Bechstein W., Waldschmidt D., Jacobasch L., Wilhelm M., Rau B.M. (2017). CONKO-005: Adjuvant Chemotherapy With Gemcitabine Plus Erlotinib Versus Gemcitabine Alone in Patients After R0 Resection of Pancreatic Cancer: A Multicenter Randomized Phase III Trial. J. Clin. Oncol..

[7] Han S.S., Jang J.Y., Kim S.W., Kim W.H., Lee K.U., Park Y.H. (2006). Analysis of long-term survivors after surgical resection for pancreatic cancer. Pancreas.

[8] Ferrone C.R., Brennan M.F., Gonen M., Coit D.G., Fong Y., Chung S., Tang L., Klimstra D., Allen P.J. (2008). Pancreatic adenocarcinoma: The actual 5-year survivors. J. Gastrointest Surg..

[9] Adham M., Jaeck D., Le Borgne J., Oussoultzouglou E., Chenard-Neu M.P., Mosnier J.F., Scoazec J.Y., Mornex F., Partensky C. (2008). Long-term survival (5-20 years) after pancreatectomy for pancreatic ductal adenocarcinoma: A series of 30 patients collected from 3 institutions. Pancreas.

[10] Ferrone C.R., Pieretti-Vanmarcke R., Bloom J.P., Zheng H., Szymonifka J., Wargo J.A., Thayer S.P., Lauwers G.Y., Deshpande V., Mino-Kenudson M. (2012). Pancreatic ductal adenocarcinoma: Long-term survival does not equal cure. Surgery.

[11] Biankin A.V., Waddell N., Kassahn K.S., Gingras M.C., Muthuswamy L.B., Johns A.L., Miller D.K., Wilson P.J., Patch A.M., Wu J. (2012). Pancreatic cancer genomes reveal aberrations in axon guidance pathway genes. Nature.

[12] McIntyre C.A., Lawrence S.A., Richards A.L., Chou J.F., Wong W., Capanu M., Berger M.F., Donoghue M.T.A., Yu K.H., Varghese A.M. (2020). Alterations in driver genes are predictive of survival in patients with resected pancreatic ductal adenocarcinoma. Cancer.

[13] Bailey P., Chang D.K., Nones K., Johns A.L., Patch A.M., Gingras M.C., Miller D.K., Christ A.N., Bruxner T.J., Quinn M.C. (2016). Genomic analyses identify molecular subtypes of pancreatic cancer. Nature.

[14] Pentheroudakis G., Committee E.G. (2019). Recent eUpdates to the ESMO Clinical Practice Guidelines on hepatocellular carcinoma, cancer of the pancreas, soft tissue and visceral sarcomas, cancer of the prostate and gastric cancer. Ann. Oncol..

[15] Ducreux M., Cuhna A.S., Caramella C., Hollebecque A., Burtin P., Goere D., Seufferlein T., Haustermans K., Van Laethem J.L., Conroy T. (2015). Cancer of the pancreas: ESMO Clinical Practice Guidelines for diagnosis, treatment and follow-up. Ann. Oncol..

[16] Raphael B.J., Hruban R.H., Aguirre A.J., Moffitt R.A., JenYeh J., Stewart C., GordonRobertson A., Cherniack A.D., Gupta M., Cancer Genome Atlas Research Network (2017). Integrated Genomic Characterization of Pancreatic Ductal Adenocarcinoma. Cancer Cell.

[17] Bernard V., Semaan A., Huang J., San Lucas F.A., Mulu F.C., Stephens B.M., Guerrero P.A., Huang Y., Zhao J., Kamyabi N. (2019). Single-Cell Transcriptomics of Pancreatic Cancer Precursors Demonstrates Epithelial and Microenvironmental Heterogeneity as an Early Event in Neoplastic Progression. Clin. Cancer Res..

[18] Fischer C.G., Wood L.D. (2018). From somatic mutation to early detection: Insights from molecular characterization of pancreatic cancer precursor lesions. J. Pathol..

[19] Maitra A., Adsay N.V., Argani P., Iacobuzio-Donahue C., De Marzo A., Cameron J.L., Yeo C.J., Hruban R.H. (2003). Multicomponent analysis of the pancreatic adenocarcinoma progression model using a pancreatic intraepithelial neoplasia tissue microarray. Mod. Pathol..

[20] Murphy S.J., Hart S.N., Lima J.F., Kipp B.R., Klebig M., Winters J.L., Szabo C., Zhang L., Eckloff B.W., Petersen G.M. (2013). Genetic alterations associated with progression from pancreatic intraepithelial neoplasia to invasive pancreatic tumor. Gastroenterology.

[21] Visani M., Acquaviva G., De Leo A., Sanza V., Merlo L., Maloberti T., Brandes A.A., Franceschi E., Di Battista M., Masetti M. (2021). Molecular alterations in pancreatic tumors. World J. Gastroenterol..

[22] Luchini C., Brosens L.A.A., Wood L.D., Chatterjee D., Shin J.I., Sciammarella C., Fiadone G., Malleo G., Salvia R., Kryklyva V. (2021). Comprehensive characterisation of pancreatic ductal adenocarcinoma with microsatellite instability: Histology, molecular pathology and clinical implications. Gut.

[23] Waddell N., Pajic M., Patch A.M., Chang D.K., Kassahn K.S., Bailey P., Johns A.L., Miller D., Nones K., Quek K. (2015). Whole genomes redefine the mutational landscape of pancreatic cancer. Nature.

[24] Masetti M., Acquaviva G., Visani M., Tallini G., Fornelli A., Ragazzi M., Vasuri F., Grifoni D., Di Giacomo S., Fiorino S. (2018). Long-term survivors of pancreatic adenocarcinoma show low rates of genetic alterations in KRAS, TP53 and SMAD4. Cancer Biomark.

[25] Oshima M., Okano K., Muraki S., Haba R., Maeba T., Suzuki Y., Yachida S. (2013). Immunohistochemically detected expression of 3 major genes (CDKN2A/p16, TP53, and SMAD4/DPC4) strongly predicts survival in patients with resectable pancreatic cancer. Ann. Surg..

[26] Kang C.M., Hwang H.K., Park J., Kim C., Cho S.K., Yun M., Lee W.J. (2016). Maximum Standard Uptake Value as a Clinical Biomarker for Detecting Loss of SMAD4 Expression and Early Systemic Tumor Recurrence in Resected Left-Sided Pancreatic Cancer. Medicine.

[27] Amin M.B., American Joint Committee on Cancer, American Cancer Society (2017). AJCC Cancer Staging Manual.

[28] de Biase D., Acquaviva G., Visani M., Sanza V., Argento C.M., De Leo A., Maloberti T., Pession A., Tallini G. (2020). Molecular Diagnostic of Solid Tumor Using a Next Generation Sequencing Custom-Designed Multi-Gene Panel. Diagnostics.

[29] Barugola G., Partelli S., Marcucci S., Sartori N., Capelli P., Bassi C., Pederzoli P., Falconi M. (2009). Resectable pancreatic cancer: Who really benefits from resection?. Ann. Surg. Oncol..

[30] Guo S.W., Shen J., Gao J.H., Shi X.H., Gao S.Z., Wang H., Li B., Yuan W.L., Lin L., Jin G. (2020). A preoperative risk model for early recurrence after radical resection may facilitate initial treatment decisions concerning the use of neoadjuvant therapy for patients with pancreatic ductal adenocarcinoma. Surgery.

[31] Kimura K., Amano R., Nakata B., Yamazoe S., Hirata K., Murata A., Miura K., Nishio K., Hirakawa T., Ohira M. (2014). Clinical and pathological features of five-year survivors after pancreatectomy for pancreatic adenocarcinoma. World J. Surg. Oncol..

[32] La Torre M., Nigri G., Lo Conte A., Mazzuca F., Tierno S.M., Salaj A., Marchetti P., Ziparo V., Ramacciato G. (2014). Is a preoperative assessment of the early recurrence of pancreatic cancer possible after complete surgical resection?. Gut Liver.

[33] Suzuki S., Shimoda M., Shimazaki J., Maruyama T., Oshiro Y., Nishida K., Sahara Y., Nagakawa Y., Tsuchida A. (2018). Predictive Early Recurrence Factors of Preoperative Clinicophysiological Findings in Pancreatic Cancer. Eur. Surg. Res..

[34] Mosele F., Remon J., Mateo J., Westphalen C.B., Barlesi F., Lolkema M.P., Normanno N., Scarpa A., Robson M., Meric-Bernstam F. (2020). Recommendations for the use of next-generation sequencing (NGS) for patients with metastatic cancers: A report from the ESMO Precision Medicine Working Group. Ann. Oncol..

[35] Hayashi H., Kohno T., Ueno H., Hiraoka N., Kondo S., Saito M., Shimada Y., Ichikawa H., Kato M., Shibata T. (2017). Utility of Assessing the Number of Mutated KRAS, CDKN2A, TP53, and SMAD4 Genes Using a Targeted Deep Sequencing Assay as a Prognostic Biomarker for Pancreatic Cancer. Pancreas.

[36] Gillson J., Ramaswamy Y., Singh G., Gorfe A.A., Pavlakis N., Samra J., Mittal A., Sahni S. (2020). Small Molecule KRAS Inhibitors: The Future for Targeted Pancreatic Cancer Therapy?. Cancers.

[37] Luchini C., Paolino G., Mattiolo P., Piredda M.L., Cavaliere A., Gaule M., Melisi D., Salvia R., Malleo G., Shin J.I. (2020). KRAS wild-type pancreatic ductal adenocarcinoma: Molecular pathology and therapeutic opportunities. J. Exp. Clin. Cancer Res..

[38] Crino S.F., Di Mitri R., Nguyen N.Q., Tarantino I., de Nucci G., Deprez P.H., Carrara S., Kitano M., Shami V.M., Fernandez-Esparrach G. (2021). Endoscopic Ultrasound-guided Fine-needle Biopsy With or Without Rapid On-site Evaluation for Diagnosis of Solid Pancreatic Lesions: A Randomized Controlled Non-Inferiority Trial. Gastroenterology.

[39] Fabbri C., Fornelli A., Fuccio L., Giovanelli S., Tarantino I., Antonini F., Liotta R., Frazzoni L., Gusella P., La Marca M. (2019). High diagnostic adequacy and accuracy of the new 20G procore needle for EUS-guided tissue acquisition: Results of a large multicentre retrospective study. Endosc. Ultrasound..

[40] Larghi A., Lawlor R.T., Crino S.F., Luchini C., Rizzatti G., Curatolo M., Gabbrielli A., Inzani F., Scarpa A. (2020). Endoscopic ultrasound guided fine needle biopsy samples to drive personalized medicine: A proof of concept study. Pancreatology.

